# The Epidemiology of Skin Cancer in Queensland: The Significance of Premalignant Conditions

**DOI:** 10.1038/bjc.1961.52

**Published:** 1961-09

**Authors:** G. G. Carmichael


### THE EPIDEMIOLOGY OF SKIN CANCER IN QUEENSLAND: THE SIGNIFICANCE OF PREMALIGNANT CONDITIONS

G. G. CARMICHAEL*

*From the University of Queensland Medical School, Brisbane*

Received for publication June 27, 1961

In a previous paper (Carmichael and Silverstone, 1961) a method of calculating the incidence rates of skin cancer in four Queensland coastal cities, based on hospital records was described. In this paper these same records are analysed further with respect to type and site of lesions exhibited by the patients. Only the records of patients giving addresses in Brisbane, Rockhampton, Townsville and Cairns are used, so that uniformity of some of the variables is ensured.

The concept of premalignancy is, in the main, a clinical and pathological one, though it gets some assistance from statistics in a few instances.

The statistical approach to premalignancy is essentially one of testing the association of attributes. If the proportion of people suffering from a pathological condition $X$ in a general population is less than the proportion in a population also suffering from a cancer $Y$, then $X$ and $Y$ are positively associated, and $X$ could be related to the genesis of the cancer. The conclusions drawn from this type of argument would be influenced by such considerations as the relative frequency of the two conditions, the prognosis of the cancer, and whether the presence of the precancerous lesion merits treatment to prevent the malignancy supervening.

It is usual to consider hyperkeratosis as premalignant for both squamous and basal cell cancer (Belisario, 1959; Payling Wright, 1958), though hyperkeratosis is more nearly related to squamous cell cancer histologically. Hyperkeratosis, or solar keratosis as it is often called, may also remain a benign lesion.

In Queensland both skin cancer and solar keratosis are very common, and it is of interest to test the association of these attributes in the samples of records already referred to.

### ANALYSIS OF THE DATA

Samples of records of patients presenting at the clinics of the Queensland Radium Institute at Brisbane, Rockhampton, Townsville and Cairns, in the ten year period 1948–1957 were classified according to whether treatment had been for squamous or basal cell carcinoma or hyperkeratosis.

The following symbols were used :—

A, $\alpha$ : Presence or absence of basal cell cancer.
B, $\beta$ : Presence or absence of squamous cell cancer.
C, $\gamma$ : Presence or absence of solar keratosis.

* Present address :—The Institute of Medical and Veterinary Science, Frome Road, Adelaide, South Australia.

There are eight possible combinations of these attributes, but only seven appear in this analysis because there is no $\alpha\beta\gamma$ population. It was possible to show that there was no difference in the proportions of the lesions developed between the four cities, though there was a difference between the sexes. The frequencies are therefore amalgamated for each city and are shown in Table I. In most cases

TABLE I.—*Classification of Patients According to Types of lesions developed. Amalgamated Data from the Four Centres. Figures in brackets are percentages*

| | Male | Female | Total |
|---|---|---|---|
| $A\beta\gamma$ | 324 (27·9) | 194 (21·5) | 518 (25·1) |
| $aB\gamma$ | 96 (8·3) | 38 (4·2) | 134 (6·5) |
| $a\beta C$ | 413 (35·5) | 465 (51·4) | 878 (42·5) |
| $AB\gamma$ | 21 (1·8) | 7 (0·8) | 28 (1·4) |
| $A\beta C$ | 206 (17·7) | 151 (16·7) | 357 (17·3) |
| $aBC$ | 43 (3·7) | 33 (3·7) | 76 (3·7) |
| $ABC$ | 60 (5·2) | 16 (1·8) | 76 (3·7) |
| Total | 1163 | 904 | 2067 |

the diagnosis is a clinical one, only atypical lesions being examined histologically. The solar keratosis is a lesion that may vary from a thin keratinous plaque to a thickened hyperkeratotic area that may measure up to a centimetre in diameter.

If the frequencies in each classification are tabulated, certain inequalities are apparent. The direction of the inequalities is the same for the males as for the females, though the numbers are different. Brackets indicate frequencies.

$$(A\beta\gamma) > (A\beta C)$$
$$(\alpha B\gamma) > (\alpha BC)$$
$$(A\beta\gamma) + (A\beta C) > (AB\gamma) + (ABC)$$
$$(\alpha B\gamma) + (\alpha BC) > (AB\gamma) + (ABC)$$
$$(AB\gamma) < (ABC)$$

Any *a priori* hypothesis of premalignancy must be arbitrary, and cannot be tested statistically with any meaning. However, it is reasonable to test for independence, and to take note of divergences from expectation. Three two-way tables are relevant for the associations between the two forms of cancer and hyperkeratosis, and these are set out in Tables II, III and IV. The expected values on the basis of independence are given in brackets.

TABLE II.—*The Association Between Basal Cell Cancer and Solar Keratosis*

| | Male | | | | Female | | |
|---|---|---|---|---|---|---|---|
| | A | a | Total | | A | a | Total |
| C | 266 | 456 | 722 | C | 167 | 498 | 665 |
| | (379·3) | (342·7) | | | (270·7) | (394·3) | |
| $\gamma$ | 345 | 96 | 441 | $\gamma$ | 201 | 38 | 239 |
| | (231·7) | (209·3) | | | (97·3) | (141·7) | |
| Total | 611 | 552 | 1163 | Total | 368 | 536 | 904 |

Except for the association between the skin cancers occurring together in the presence of hyperkeratosis in the case of females (Table IV), there is marked divergence from expectation on the hypothesis of independence. The $\chi^2$ values

TABLE III.—*The Association Between Squamous Cell Cancer and Solar Keratosis*

| | Male | | | | Female | | |
|---|---|---|---|---|---|---|---|
| | B | $\beta$ | Total | | B | $\beta$ | Total |
| C | 103 (136·6) | 619 (584·4) | 722 | C | 49 (69·1) | 616 (595·9) | 665 |
| $\gamma$ | 117 (83·4) | 324 (357·6) | 441 | $\gamma$ | 45 (24·9) | 194 (214·1) | 239 |
| Total | 220 | 943 | 1163 | Total | 94 | 810 | 904 |

TABLE IV.—*The Association Between Basal and Squamous Cell Cancer in the presence of Keratosis*

| | Male | | | | Female | | |
|---|---|---|---|---|---|---|---|
| | AC | $\alpha$C | Total | | AC | $\alpha$C | Total |
| BC | 60 (37·9) | 43 (65·1) | 103 | BC | 16 (12·3) | 33 (36·7) | 49 |
| $\beta$C | 206 (228·1) | 413 (390·9) | 619 | $\beta$C | 151 (154·7) | 465 (461·3) | 616 |
| Total | 266 | 456 | 722 | Total | 167 | 498 | 665 |

are well outside the limits expected by chance, except for the case of the females mentioned, which has a value of 1·6042 ($0{\cdot}3 > P > 0{\cdot}2$). Between each type of skin cancer occurring separately and hyperkeratosis there is dissociation. This is not unexpected on clinical grounds for basal cell cancer, but it was anticipated that there would be a positive association between squamous cell cancer and hyperkeratosis. The patients represented in Table IV include those who are acutely sensitive to the action of ultraviolet radiation, suffer from severe solar dermatitis and develop multiple lesions.

In interpreting these findings three possibilities arise that may explain them :

(i) the patients tend only to present with and be treated for skin cancer, hyperkeratosis being largely neglected ;
(ii) treatment of hyperkeratosis prevents the subsequent development of skin cancer, and
(iii) the period of observation was not long enough.

Regarding the first possibility it will be noted that 35·5 per cent of the males and 51·4 per cent of the females are in the series for hyperkeratosis alone. The notes are well kept, and treatment for hyperkeratosis is often with superficial X-ray, which necessitates the lesions being accurately described. Concerning (ii) it should be borne in mind that solar keratoses are frequently multiple and that if a positive association exists between squamous cell cancer and keratosis the cancer patients should also show the premalignant lesion. About the third possibility, the mean time of observation was five years, which should give time for multiple lesions to declare themselves and some to develop into malignancy. It would appear that the data and statistics presented are an accurate description of the patients presenting for treatment.

The anatomical distribution of the lesions also throws light on the question. In Table V are set out the distribution of the lesions in those patients who have had only one type of lesion. It will be seen that the two cancers tend to have their own sites of predilection, and that keratosis occurs with roughly equal

TABLE V.—*The Anatomical Distribution of Lesions in Selected Sites, in Patients who have been Treated for only One Type of Lesion. Figures in brackets are percentages*

MALE

| | B.C.C. | S.C.C. | Keratosis |
|---|---|---|---|
| Forehead and temple | 58 (12·1) | 5 (4·8) | 83 (11·2) |
| Periorbital region | 59 (12·3) | 5 (4·8) | 28 (3·8) |
| Malar region | 111 (23·1) | 6 (5·7) | 122 (16·5) |
| Nose | 88 (18·3) | 10 (9·5) | 130 (17·6) |
| Ear, anterior surface | 20 (4·2) | 11 (10·5) | 52 (7·0) |
| Neck | 35 (7·3) | 7 (6·7) | 23 (3·1) |
| Forearm | 14 (2·9) | 16 (15·2) | 81 (11·0) |
| Hand | 6 (1·2) | 18 (17·1) | 125 (16·9) |
| Total | 391 (81·4) | 78 (74·3) | 644 (87·1) |

FEMALE

| | B.C.C. | S.C.C. | Keratosis |
|---|---|---|---|
| Forehead and temple | 38 (14·8) | 5 (12·5) | 72 (9·1) |
| Periorbital region | 24 (9·3) | 2 (5·0) | 32 (4·0) |
| Malar region | 43 (16·7) | 8 (20·0) | 172 (21·7) |
| Nose | 67 (26·1) | 4 (10·0) | 197 (24·8) |
| Ear, anterior surface | 4 (1·5) | 1 (2·5) | 5 (0·6) |
| Neck | 15 (5·8) | 3 (7·5) | 15 (1·9) |
| Forearm | 7 (2·7) | 5 (12·5) | 80 (10·1) |
| Hand | 3 (1·2) | 8 (20·0) | 143 (18·0) |
| Total | 201 (78·1) | 36 (90·0) | 716 (90·2) |

frequency in areas of predilection of both cancers. There are some exceptions to this. For instance, in the neck the cancers are relatively frequent, but only a small proportion of the keratoses occur in this site. The suggestion is that the malignancies do not evolve through a premalignant stage.

### SUMMARY AND CONCLUSIONS

Data are presented for four cities in Queensland that show that there is a tendency for both basal and squamous cell cancer to occur in the absence of hyperkeratosis. The converse also holds. The anatomical distribution of the keratoses does not follow that of one particular form of cancer, but roughly that of both, although basal cell cancers and hyperkeratoses are not likely to be confused clinically. It seems that solar keratosis is not an important premalignant lesion, but rather that it occurs independently.

In this, as in the preceding paper on the incidence of skin cancer, it must be stressed that geographical factors are specially important, and that what is true for Queensland is not necessarily true for other regions further from the Equator.

This work was carried out during the tenure of a Medical Research Fellowship at the University of Queensland. I would like to acknowledge the advice and help of Dr. A. G. S. Cooper, Director of the Queensland Radium Institute.

### REFERENCES

BALISARIO, J. C.—(1959) ‘ Cancer of the Skin ’. London (Butterworth).
CARMICHAEL, G. G. AND SILVERSTONE, H.—(1961) *Brit. J. Cancer*, **15**, 409.
PAYLING WRIGHT, G.—(1958) ‘ An Introduction to Pathology,’ 3rd edition. London Longmans).


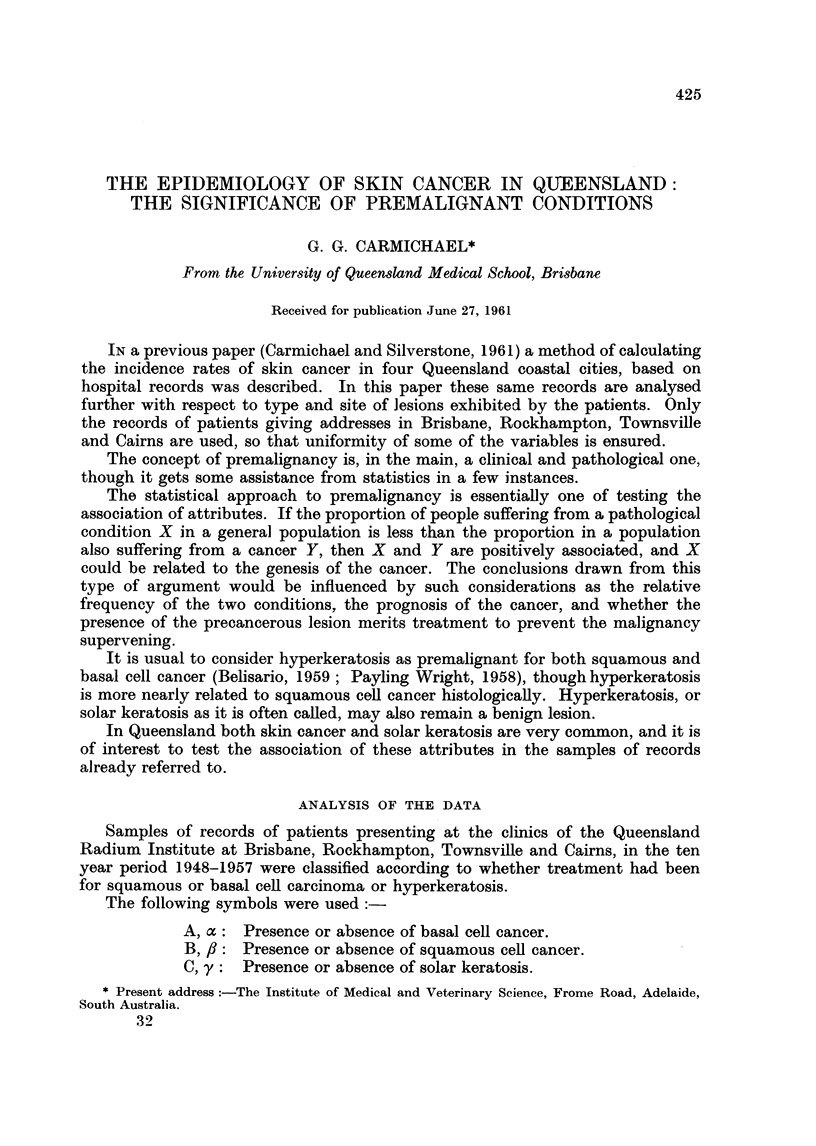

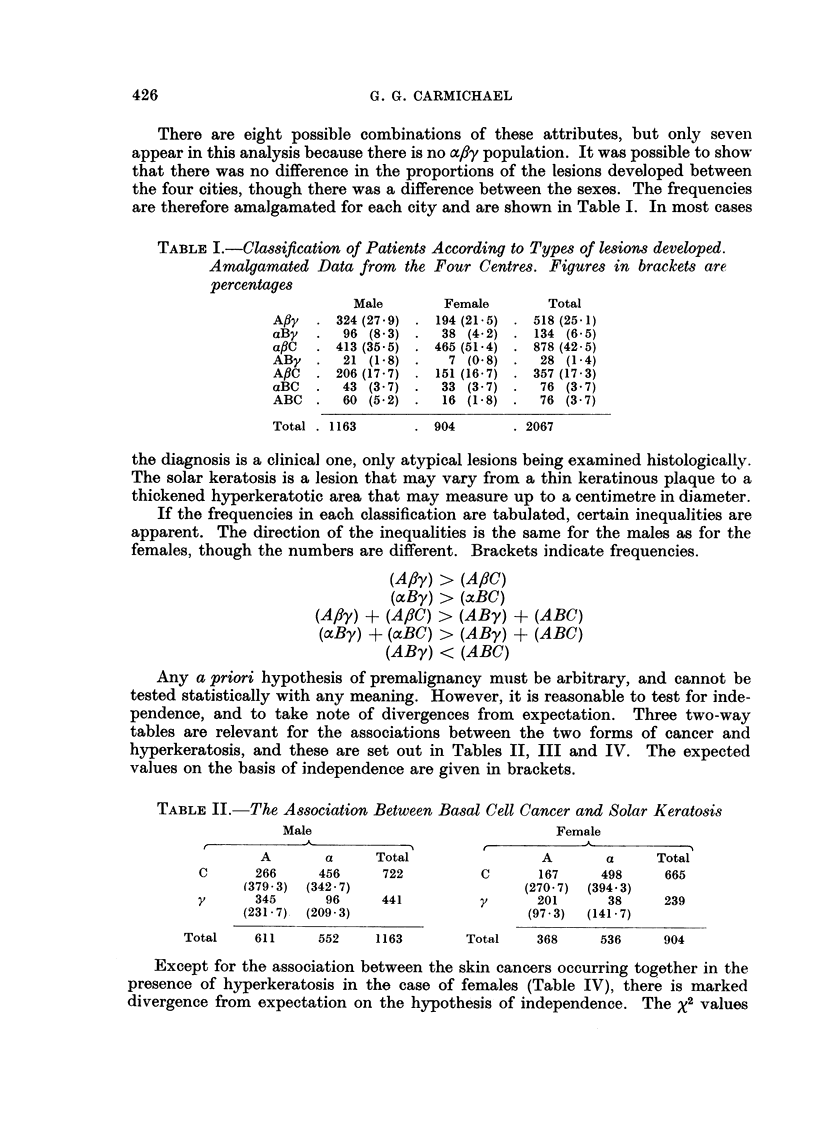

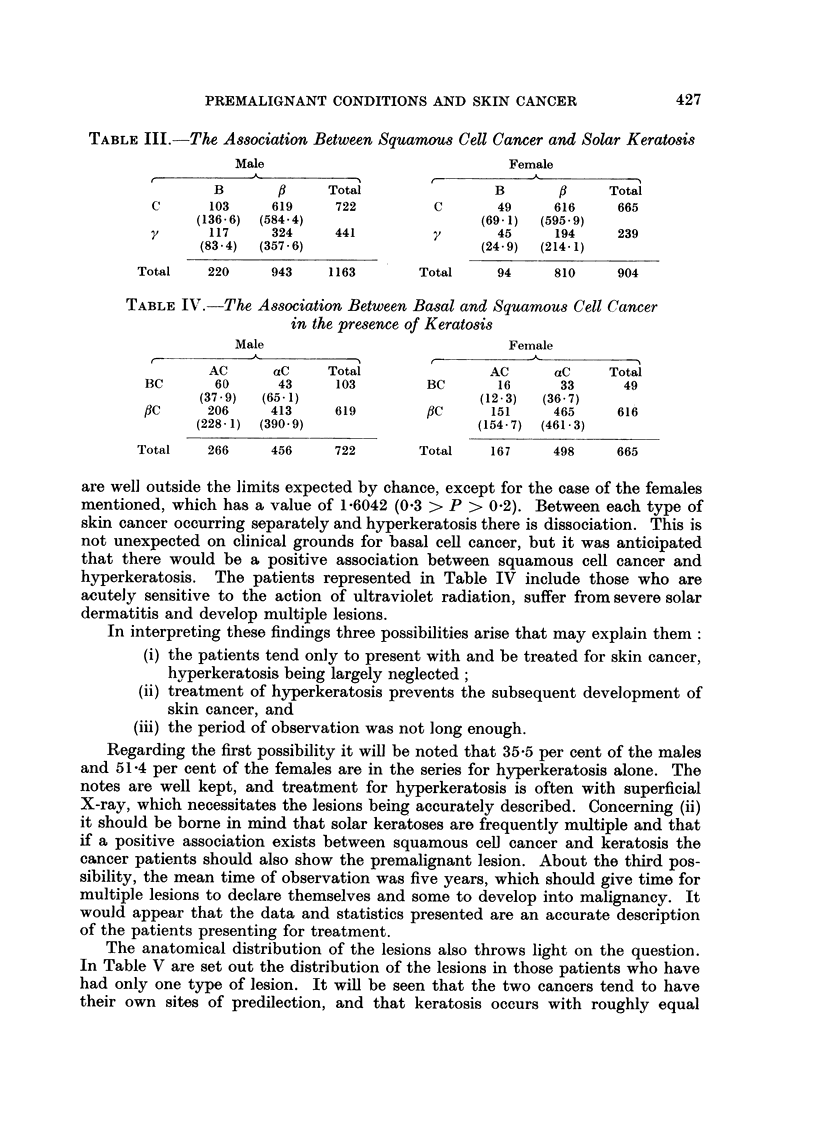

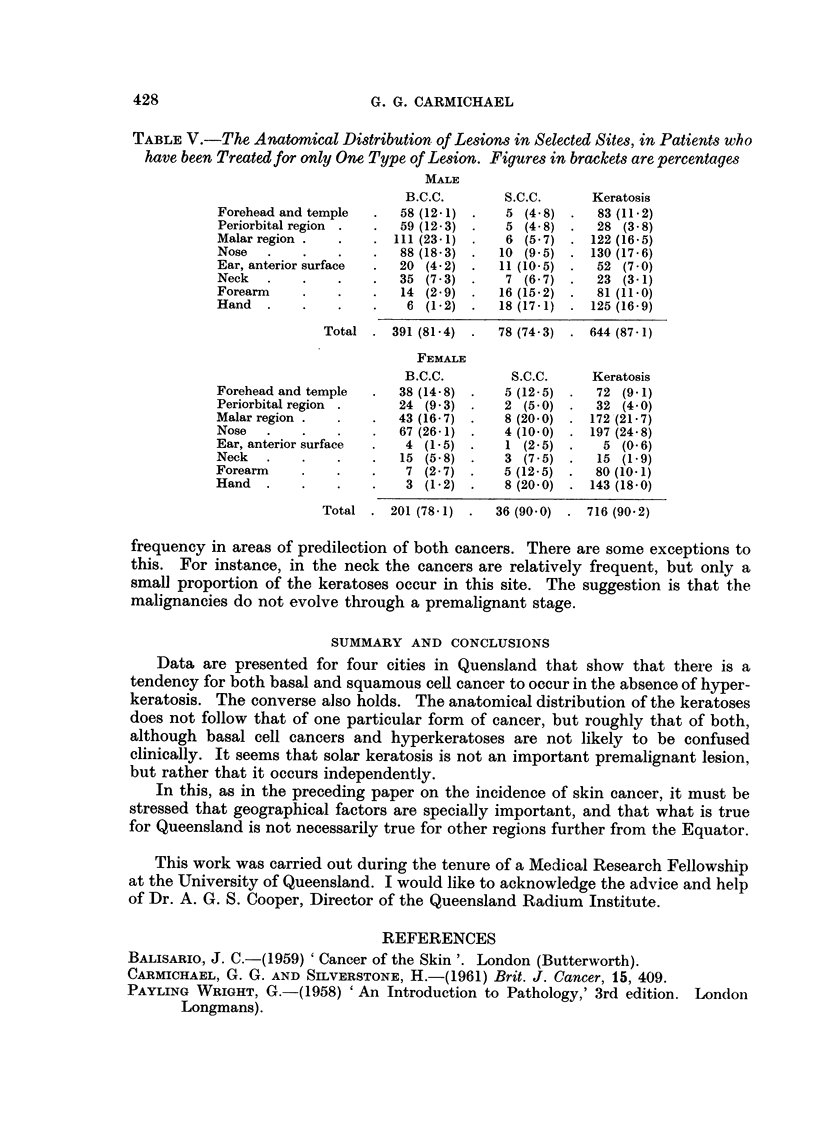


## References

[OCR_00326] CARMICHAEL G. G., SILVERSTONE H. (1961). The epidemiology of skin cancer in Queensland: the incidence.. Br J Cancer.

